# Health services and programmatic responses to improving adolescent HIV care in Lusaka, Zambia: A descriptive qualitative study

**DOI:** 10.4102/phcfm.v18i1.5189

**Published:** 2026-02-17

**Authors:** Kaala Moomba, Talitha Crowley, Brian van Wyk

**Affiliations:** 1 School of Public Health, Faculty of Community and Health Sciences, University of the Western Cape, Bellville, South Africa; 2 School of Nursing, Faculty of Community and Health Sciences, University of the Western Cape, Bellville, South Africa

**Keywords:** adolescents, antiretroviral therapy, cohort, viral suppression, retrospective, Zambia

## Abstract

**Background:**

Adolescents living with human immunodeficiency virus (HIV) face disproportionately poor treatment outcomes because of a combination of psychosocial, structural and health systems challenges. Despite efforts to implement adolescent-friendly and differentiated service delivery models in Zambia, gaps remain in the organisation and delivery of care.

**Aim:**

This study aimed to explore how HIV services for adolescents are delivered and experienced by healthcare workers and programme managers in Lusaka District.

**Setting:**

The study was conducted across six health facilities in Lusaka District, Zambia, representing different levels of the health system and providing HIV care to adolescents.

**Methods:**

A descriptive qualitative design was employed. Thirty purposively selected participants (24 healthcare workers and six HIV programme managers) participated in individual in-depth interviews (IDIs) conducted between 20 February 2025 and 30 April 2025 using a semi-structured interview guide. Data were audio-recorded, transcribed verbatim and analysed using inductive content analysis.

**Results:**

Inductive content analysis identified three themes: programmatic responses, highlighting strategic leadership, policy direction, adolescent-focused service delivery and workforce capacity building; implementation challenges, including inadequate infrastructure, health system limitations and individual-level barriers; and opportunities for improved programmatic response and service delivery, emphasising improvements in policy, health systems, infrastructure and service delivery.

**Conclusion:**

Although notable progress has been made in expanding adolescent HIV services in Lusaka, gaps in infrastructure, workforce and social support continue to affect programme effectiveness. Strengthening adolescent-responsive and sustainable care is essential to improve retention, viral suppression and Zambia’s progress towards the Joint United Nations Programme on HIV/AIDS (UNAIDS) 95-95-95 targets in the adolescent HIV cascade.

**Contribution:**

This study highlights barriers and opportunities in adolescent HIV programming in Lusaka, emphasising leadership, policy, service delivery and workforce capacity, while noting ongoing system and individual challenges. The findings can inform policy and practice to strengthen adolescent-responsive HIV care in Zambia and similar contexts.

## Introduction

Adolescents living with human immunodeficiency virus (ALHIV) represent a growing and vulnerable population within the global HIV epidemic.^1^ As of 2022, approximately 1.7 million adolescents (aged 10–19 years) were living with HIV worldwide, with sub-Saharan Africa bearing the greatest burden.^2^ Despite substantial progress in antiretroviral therapy (ART) scale-up, adolescents remain at increased risk of poor treatment outcomes compared to other age groups.^1^ Challenges such as suboptimal adherence, loss to follow-up and virologic failure are disproportionately more common among ALHIV, posing significant barriers to achieving optimal treatment outcomes. These challenges are often driven by a complex interplay of psychosocial, socioeconomic and health system factors.^1,3,4^ Psychosocial barriers include stigma, fear of disclosure, mental health conditions such as depression and anxiety and limited social support, which negatively impact motivation for persistent medication use.^5,6^ Socioeconomic challenges such as poverty, long distances to health facilities and competing priorities with school attendance further constrain access and engagement in care.^7^ In addition, health system factors such as lack of adolescent-friendly services, inadequate provider training, high staff turnover and insufficient integration of mental health and HIV services undermine continuity of care and trust in the healthcare system.^1,8,9^ Collectively, these barriers contribute to higher rates of poor adherence and viral non-suppression among ALHIV compared to other age groups on HIV treatment.^10,11,12^ The combination of these factors underscores the urgent need for comprehensive, adolescent-centred and contextually appropriate interventions in sub-Saharan Africa.

In Zambia, the prevalence of HIV among adolescents and young people (AYP) is estimated at 3.8%, with those aged 15–19 years accounting for 1.9%.^13^ Improving access to and retention in ART care for this age group is recognised as a national priority.^14,15^ The Ministry of Health, in collaboration with partners, has implemented differentiated service delivery (DSD) models and adolescent-friendly health services to better address the unique needs of this group.^16^ However, despite these innovations, gaps persist in service delivery and support programming, particularly regarding how care is organised and delivered at the facility and programmatic levels.

In Zambia, national efforts to strengthen adolescent HIV programming have emphasised strategic leadership and policy direction through technical working groups (TWGs) and frameworks such as the Zambia Consolidated Guidelines, the National Adolescent Health Strategic Plan and Adolescent-Friendly Health Services Standards, aimed at aligning implementation with national and global priorities.^17,18,19^ In addition, adolescent-focused service delivery models, including DSD approaches such as dedicated clinic days, youth-friendly spaces, multi-month ART refills and fast-track systems, have been introduced to improve convenience, reduce stigma and enhance engagement in care.^20,21,22^ The integration of sexual and reproductive health (SRH) services into HIV care has further sought to address adolescents’ comprehensive health needs in line with best practices for adolescent-responsive services,^23,24^ while the establishment of dedicated youth-friendly spaces has aimed to create supportive and holistic service environments for ALHIV.^25^ However, how these programmatic strategies are implemented and experienced by frontline providers and programme managers remains insufficiently explored.

Despite the expansion of adolescent-focused HIV policies and service delivery models in Zambia, most existing evidence has focused on adolescent-level outcomes, barriers and interventions, with comparatively little attention to the perspectives of those responsible for implementing these programmes at facility and health system levels. In particular, there is limited qualitative evidence examining how national policies, DSD models and adolescent-friendly service standards are operationalised in routine primary health care (PHC) settings and the contextual challenges that shape their implementation. Understanding health care workers’ (HCWs’) and HIV programme managers’ experiences is critical for identifying implementation gaps, unintended consequences and opportunities for strengthening service delivery, yet such perspectives remain under-represented in the Zambian literature.

This study aimed to address this gap by exploring how HIV service delivery and support programmes for adolescents are implemented, experienced and perceived by HCWs and programme managers in Lusaka District, which serves as a key urban hub for HIV service delivery in Zambia. By generating implementation-focused, provider- and system-level insights, this study contributes contextually grounded evidence to inform the optimisation of adolescent HIV services within PHC settings.

## Research methods and design

### Study design

A qualitative descriptive design was applied to capture detailed, experience-near accounts of implementation experiences, challenges and opportunities from HCWs and HIV programme managers regarding adolescent HIV service delivery for improving care for ALHIV.

### Study setting

Lusaka is the capital city of Zambia and the country’s most populous urban centre, with an estimated population of over 3 million people.^26^ Lusaka Province comprises both urban and peri-urban districts, with Lusaka District serving as the administrative and healthcare delivery hub. Lusaka District has the highest number of adolescents receiving ART (> 75%) and has implemented several intervention packages for adolescents receiving ART, including peer support models, adolescent-focused DSD and integrated psychosocial support programmes.^27^ The district is linguistically diverse, with major languages including Nyanja, Bemba, Tonga and Lozi; however, Nyanja serves as the predominant lingua franca and is widely used in routine communication within health facilities. Adolescent services are found both at tertiary healthcare and PHC levels, with the tertiary levels offering more specialised services for complicated cases, including treatment failure, mental health conditions and co-infections. Some adolescent health services offered at PHC specifically for adolescents include HIV and acquired immunodeficiency syndrome (AIDS) and sexually transmitted infection (STI) services, alcohol and substance abuse counselling, SRH services and non-communicable disease screening. These services are supported through partnerships involving the Ministry of Health, international donors (President’s Emergency Plan for AIDS Relief [PEPFAR], Global Fund) and local implementing partners.

To make the PHC facilities more adolescent-friendly, adolescent spaces have been provided in health facilities with the introduction of specific adolescent clinic days. These spaces are often staffed by trained adolescent focal point providers and peer educators and aim to ensure confidentiality, reduce stigma and improve health-seeking behaviour among adolescents.^28^

### Study population and sampling strategy

Healthcare workers were purposively selected from six health facilities with outpatient departments providing HIV services to ALHIV on ART. A total of 30 participants were interviewed between 20 February 2025 and 30 April 2025, including 24 HCWs and six HIV programme managers. The HCWs were drawn from four PHC facilities and two first-level (district) hospitals and comprised 18 females and six males. Recruitment included all HCWs who were currently involved in the care of ALHIV. The health facilities were selected because they serve high numbers of adolescents on ART, ensuring inclusion of HCWs with substantial experience in ALHIV care, with one hospital additionally chosen as a centre of excellence for paediatric and adolescent services providing specialised care. One participant who withdrew because of discomfort was replaced. Human immunodeficiency virus programme managers represented different levels of the health system, namely: Ministry of Health headquarters (1), district health office (1), provincial health office (2) and non-governmental organisations (2) (Table 1) and were included based on their current managerial roles and experience in HIV and/or ALHIV programming.

**TABLE 1:** Summary characteristics of participating healthcare workers and human immunodeficiency virus programme managers.

Participant characteristics	Female	Male	Total
**Healthcare workers**			
Medical doctor	1	0	1
HIV nurse practitioners	8	0	8
Nurse (general registered nurse)	5	2	7
Clinical officers	0	2	2
Psychosocial counsellors	3	2	5
Pharmacists	0	1	1
Sub-total	-	-	24
**HIV programme managers**			
Non-governmental organisation	2	0	2
Ministry of Health	0	1	1
Provincial health office	1	1	2
District health office	1	0	1
Sub-total	-	-	6

**Total**	-	-	**30**

HIV, human immunodeficiency virus.

### Data collection

Face-to-face in-depth interviews (IDIs) were conducted using semi-structured interview guides, with a total of 30 IDIs conducted, including 24 HCWs and six HIV programme managers (Table 1). The interview guides were not adapted from a single model but were systematically developed based on the study research question, preliminary quantitative analyses of determinants of viral suppression and retention in care among ALHIV and a review of national policies and guidelines for ALHIV management, reflecting established individual, service and system-level domains. Interview guides were initially developed in English, translated into Nyanja and back-translated to ensure accuracy. A pilot was conducted in one facility to refine clarity. Interviews were conducted in either English or Nyanja, depending on participant preference. The first author conducted all interviews, took notes during sessions and ensured informed consent was obtained. The interviews were conducted in a private and secure room in the health facilities for HCWs and in a private and secure room in the office premises for HIV programme managers at times convenient to participants during working hours. Recruitment of HCWs was done through the health facility managers, while that of the HIV programme managers was done directly by the first author. Once they agreed to participate in the interview, the first author obtained informed consent by guiding participants through the information sheet and obtaining their written consent through signing a consent form. All IDIs, which lasted an average of 30 min – 35 min, were audio-recorded, transcribed verbatim, and those conducted in Nyanja were translated into English by the first author to maintain consistency and accuracy.

### Data analysis

The interview transcripts were uploaded into ATLAS.ti version 24 software, and we performed inductive content analysis.^29,30^

The first author conducted the initial coding of the IDIs, which was subsequently reviewed and validated by the co-authors. Data collection and analysis were conducted concurrently, allowing preliminary insights from ongoing analysis to inform continued data collection and analytic focus. The analysis followed an iterative process, enabling continual refinement of codes and themes as data were examined. An inductive content analysis approach was employed, which is well-suited for exploring qualitative data without the constraints of a predetermined theoretical framework.^31^ This method allowed for the identification of patterns and themes to emerge from the participants’ narratives, ensuring that the findings were grounded in the data itself.

The first author first reviewed eight HCW transcripts and two HIV Programme Manager transcripts (30% of all transcripts) to develop the codebook. This early analytic phase enabled the identification of recurring concepts and informed subsequent interviews by sensitising the researcher to emerging areas of inquiry, although no formal changes were made to the sampling strategy. The transcripts and codebook were reviewed by the second and third authors. A comprehensive codebook was created through an iterative engagement process among the three authors, resulting in agreed-upon codes with definitions by consensus. To ensure accuracy, the researchers re-read and cross-checked the transcribed data against the developed codes. The data were then organised through pattern coding, whereby codes were grouped into subthemes, then into broader themes. The data were captured in an Excel table structured into three columns: themes, subthemes and codes. Data saturation, defined as the point of information redundancy, where no new themes or codes emerge, was achieved when additional interviews at the individual level yielded no new themes or subthemes.^32^ A final analytical matrix was developed to organise the key themes, subthemes and codes describing programmatic responses, implementation challenges and opportunities for improved programmatic response and service delivery targeting ALHIV as described by HCWs and HIV programme managers (Table 2). Themes under programmatic responses included: strategic leadership, policy direction, adolescent-focused service delivery models and health workforce capacity building. Themes under implementation challenges included: inadequate infrastructure, health systems constraints and individual-level barriers. Lastly, themes under opportunities for improved service delivery included: health system and infrastructure, policy and service delivery.

**TABLE 2:** Programmatic responses, implementation challenges and opportunities for improved programmatic response and service delivery targeting adolescents living with human immunodeficiency virus.

Thematic domain	Theme	Sub-theme	Code
1. Programmatic responses	1.1. Strategic leadership	1.1.1. Governance and coordination of multisectoral response	Technical working groups Sub-national implementation oversight Development of adolescent HIV policies and guidelines
	1.2. Policy direction	1.2.1. Strategic alignment	Alignment of services with national health goals and global targets
		1.2.2. Data and monitoring	Adolescent-specific indicators in the Health Management Information System (HMIS) Tracking of adolescent outcomes Routine performance reviews eHealth systems for tracking adolescents Service quality assessments
		1.2.3. Multisectoral collaboration	Coordinating across ministries Donor and partner coordination
		1.2.4. Youth engagement mandates	Inclusion of adolescents in decision-making National-provincial collaboration on youth-inclusive platforms
	1.3. Adolescent-focused service delivery models	1.3.1. Adolescent-friendly services	Dedicated adolescent clinic days Youth-friendly corners Integrated health services (SRH-HIV) HIV testing and counselling STI screening and treatment Adolescent service coordinators
		1.3.2. Differentiated service delivery (DSD) models for adolescents	Multi-month drug dispensation Fast-track Flexible appointment scheduling Weekend ART services Outreach (community and school-health services)
		1.3.3. Psychosocial support	Mental-emotional well-being Disclosure Adherence counselling Spiritual support Support groups Educational support programmes Orphans and vulnerable children support
	1.4. Health workforce capacity building	1.4.1. National training curricula	Standardised training materials (healthcare workers and community staff packages)
		1.4.2. Mentorship and support	On-site mentorship
2. Implementation challenges	2.1. Inadequate infrastructure	2.1.1. Infrastructure limitations	Limited adolescent-friendly spaces Space constraints at facilities Frequent power outages
		2.1.2. Technological limitations	System slowness (electronic health record) Inadequate hardware Poor internet reception Duplicate patient records; data inaccuracy Lack of adolescent-specific fields
	2.2. Health systems constraints	2.2.1. Human resource constraints	Shortage of trained adolescent service providers High staff turnover Lack of HCWs dedicated to adolescent-friendly space Provider stigma and bias
		2.2.2. Sustainability and funding for adolescent HIV support services	Partner withdrawal No ring-fenced government budget line Dependence on donor support
		2.2.3. Supply chain and logistics constraints	Stock-outs of essential lab commodities and HIV and STI test kits Inadequate transport for home visits or tracing adolescents
	2.3. Individual-level barriers to adherence and engagement	2.3.1. Poor adherence and engagement in care	Missed appointments Treatment interruption Low motivation for ART Competing school schedules Distance to services
		2.3.2. Stigma	Non-disclosure to peers and family Peer pressure Self-stigma
		2.3.3. Caregiver and family issues	Unsupportive guardians Lack of contact with caregivers Temporary guardianship
3. Opportunities for improved programmatic response and service delivery	3.1. Policy	3.1.1. Address policy gaps	Advocate for lower age consent laws
	3.2. Health system and infrastructure	3.2.1. Expand adolescent-friendly infrastructure	Upgrade adolescent-friendly spaces Designate adolescent only clinic areas Build additional adolescent centres of excellence
		3.2.2. Strengthen human resource capacity	Train more adolescent-specialised HCWs Retain trained staff through incentives Assign dedicated adolescent focal persons Sensitise HCWs on adolescent needs
		3.2.3. Improve logistics and supply chain management	Ensure consistent supply of lab commodities Allocate dedicated transport for follow-ups
	3.3. Service delivery	3.3.1. Enhance adolescent-centred models	Scale peer-led outreach Expand integration of SRH with HIV services Expand decentralised service points
		3.3.2. Improve adherence and engagement	Strengthen retention package Offer adolescent-specific counselling Optimise digital tools for follow-up reminders
		3.3.3. Support family and caregiver engagement	Educate guardians on adolescent care and counselling Build support networks for caregivers

HIV, human immunodeficiency virus; SRH, sexual and reproductive health; ART, antiretroviral therapy; HCW, healthcare worker; STI, sexually transmitted infection.

### Trustworthiness

The trustworthiness of the study was ensured through several measures grounded in qualitative research standards, including credibility, dependability, confirmability and transferability.^33^

Credibility was ensured by piloting the interview guides with HCWs to validate the cultural relevance and clarity of the questions, as well as through iterative questioning during interviews, which allowed for the probing and clarification of responses. Participants were reassured that there were no right or wrong answers and that their experiences were valued, which fostered openness and helped capture rich, authentic narratives.

To strengthen dependability, a transparent and systematic approach to data collection and analysis was followed. The coding process was documented thoroughly, with the first author conducting initial coding and co-authors reviewing and validating the codes and themes to promote consistency.

Confirmability was supported through the maintenance of a reflective research journal by the first author and a comprehensive audit trail documenting methodological decisions, changes and analytic processes. The first author is a health professional with experience in HIV service delivery in Zambia and is currently based in the private sector, with no employment or supervisory role in the public health facilities where the study was conducted. While this background provided contextual understanding of the health system, it also required ongoing reflexivity to minimise potential influence on data interpretation. Reflexive practices, including journaling, team-based coding and iterative analytic discussions among the authors, were used to ensure that findings remained grounded in participants’ accounts rather than researchers’ assumptions.

Transferability was addressed by providing detailed contextual descriptions of the study setting, participants and processes, enabling readers to determine the applicability of the findings to other similar contexts. Peer debriefing sessions were conducted with experienced qualitative researchers throughout the study to review emerging findings and interpretations, contributing to analytical rigour and offering alternative perspectives.

Finally, the study adhered to relevant elements of the Consolidated Criteria for Reporting Qualitative Research (COREQ) checklist to ensure transparency, completeness and methodological rigour in reporting qualitative research.^34^

### Ethical considerations

Ethical clearance to conduct this study was obtained from the University of the Western Cape Biomedical Research Ethics Committee (No. BM24/3/4), the Mulungushi University School of Medicine Ethics Committee (No. SMHS-MU2-2024-04), the Zambia National Health Research Authority (No. NHRA1186/15/05/2024) and the Zambia Ministry of Health (No. MH/101/22/3). The researchers sought verbal and written consent from all study participants (HCWs and HIV programme managers). Pseudonyms were used to identify study participants in the transcription of IDIs. In addition, participants consented to the publishing of their responses if their identities were kept anonymous. No personal information was collected from participants during the research process.

## Results

This study explored programmatic responses, implementation challenges and opportunities for improving service delivery targeting ALHIV. We categorised the findings of our study into three main thematic areas (Table 2). The first area, programmatic responses, encompassed the following themes: strategic leadership, policy direction, adolescent-focused service delivery models and capacity building, particularly in relation to the health workforce. The second area focused on implementation challenges, which included themes such as inadequate infrastructure, health system constraints and individual-level barriers to service uptake. Finally, the third area, opportunities for improved programmatic response and service delivery, highlighted key themes around strengthening health systems and infrastructure, enhancing policy frameworks and improving service delivery mechanisms.

### Thematic domain 1: Programmatic responses

#### Theme 1.1: Strategic leadership

Human immunodeficiency virus programme managers consistently identified strategic leadership as a central programmatic mechanism shaping the national adolescent HIV response. Participants highlighted the pivotal role of governance and coordination structures, particularly national-level TWGs, in steering the agenda:

‘We’ve had several partners and stakeholders working with adolescents in a very uncoordinated fashion. But in recent years, we’ve tried to bring everybody together so that we can coordinate through the technical working group.’ (P30, Male, HIV Programme Manager, Ministry of Health)

At the sub-national level, participants emphasised the importance of oversight mechanisms in promoting accountability and ensuring the effective translation of national policies into practice. These structures were described as key to facilitating the operationalisation of the adolescent HIV response.

National guidelines were frequently cited as a key reference point guiding the delivery of adolescent-specific HIV services. These documents were viewed as providing structured, standardised direction for implementation across health facilities:

‘So, the adolescent services are provided through the national guidelines that have been developed – the Zambia Consolidated Guidelines. It outlines all the areas that the provision of services needs to be made for the adolescent.’ (P30, Male, HIV Programme Manager, Ministry of Health)

In addition to national guidelines, the National Adolescent Health Strategic Plan was highlighted as a comprehensive framework guiding the scope and focus of adolescent health services. The plan was described as encompassing a wide range of priority areas to ensure a holistic approach to adolescent well-being:

‘According to the National Adolescent Strategic Plan, adolescent health services have got six pillars which include sexual and reproductive health [*SRH*], HIV and AIDS, sexually transmitted infections, sexual and gender-based violence, non-communicable diseases, alcohol and tobacco and drug use and also dealing with adolescents with special needs, including those that have disabilities.’ (P27, Female, HIV Programme Manager, Provincial Health Office)

‘The Adolescent Health Strategy describes how the services should be provided.’ (P29, Female, HIV Programme Manager, District Health Office)

The National Standards and Guidelines for Adolescent-Friendly Health Services were identified as critical for operationalising adolescent-focused care. This policy document was regarded as providing comprehensive, practical guidance on the essential components required to deliver effective, adolescent-centred services:

‘There is a policy document [*National Standards and Guidelines for Adolescent Friendly Services*] that describes how adolescent services need to be provided.’ (P30, Male, HIV Programme Manager, Ministry of Health)

#### Theme 1.2: Policy direction

Policy direction was recognised as central to strengthening adolescent HIV programming. It was described as a critical enabler for aligning service delivery with national health priorities and global commitments, including the UNAIDS 95-95-95 targets. This alignment was seen as essential for ensuring consistency, focus and measurable progress across programmatic efforts:

‘We are able to ensure that the HIV programme is implemented and rolled out in all the facilities, trying to respond to the 95-95-95 for this particular population.’ (P30, Male, HIV Programme Manager, Ministry of Health)

Strengthening data systems to better capture adolescent-specific service delivery and outcomes emerged as a key focus of programmatic efforts:

‘The Ministry of Health has now incorporated quite a number of indicators to do with young people, provision of services for the young people that have been added to the health information system.’ (P28, Male, HIV Programme Manager, Provincial Health Office)

Further, participants described the use of standard performance indicators and tools for ongoing monitoring at the facility and district levels. Regular assessments were conducted to ensure data completeness, accuracy and timeliness:

‘The Ministry of Health formulates Key Performance Indicators. The facilities assess themselves through performance assessment, which is done quarterly and the district conducts the same biannually.’ (P29, Female, HIV Programme Manager, District Health Office)

Routine performance monitoring was also highlighted as an important accountability mechanism within the adolescent HIV response. Structured review processes, including quarterly meetings, are used to assess provincial performance and promote data-driven decision-making at sub-national levels:

‘We have quarterly meetings that are held for the adolescent performance, where we review the performance of the provinces.’ (P30, Male, HIV Programme Manager, Ministry of Health)

Youth engagement mandates, including adolescent participation in decision-making and collaboration across national and provincial platforms, were recognised as key to ensuring that services remain relevant, responsive and adolescent-centred.

‘For instance, we support the Ministry annually with the youth indabas. The Ministry would organise that and then the provinces must feed in and integrate.’ (P28, Male, HIV Programme Manager, Provincial Health Office)

#### Theme 1.3: Adolescent-focused service delivery models

Adolescent-friendly services form the cornerstone of service delivery in many health facilities, with a strong emphasis on creating welcoming, responsive and tailored environments for young people. These efforts include the establishment of dedicated adolescent clinic days, youth-friendly spaces within facilities and the integration of SRH services with HIV care.

Dedicated clinic days were described as an important strategy for fostering a sense of belonging and privacy, which help reduce stigma and encourage consistent care-seeking behaviour:

‘They come for their medication on Thursdays, a specific day just for them, so they feel comfortable.’ (P22, Male, Healthcare worker, Clinical Officer)

The presence of designated youth-friendly spaces within health facilities was similarly viewed as a critical enabler for adolescent engagement. These spaces provide a recognisable and safe environment where adolescents can access services tailored to their needs:

‘When they walk in, first of all, there’s a section of the hospital which is known as the youth-friendly space.’ (P12, Female, Healthcare worker, Medical doctor)

In addition, service integration was emphasised, particularly the incorporation of SRH into routine HIV care for adolescents. Providers noted the importance of offering comprehensive services, including family planning, to address the broader health and developmental needs of this age group:

‘We’re trying to offer everything in one place; contraceptives, ART, counselling.’ (P29, Female, HIV Programme Manager, District Health Office)

Core service components also encompassed HIV testing and counselling, tuberculosis (TB) and other opportunistic infection (OI) screening, STI screening and treatment and the appointment of adolescent service coordinators to ensure focused attention to this population:

‘If there’s a TB case, we start them on treatment. If there are other OIs, we refer accordingly. We also screen for STIs and manage them accordingly. Our aim is to offer holistic care.’ (P05, Male, Healthcare worker, Clinical Officer)

To better meet the unique needs of ALHIV, health facilities implemented a range of DSD models to reduce structural barriers and improve retention.

The models included multi-month drug dispensing, which reduces the frequency of clinic visits and improves convenience for stable adolescents. Fast-track refill models enable clients to collect medication with minimal waiting time, while flexible appointment scheduling allows for services outside standard clinic hours to accommodate school and work commitments:

‘And then those that are stable, we give them drugs for six months, so that they don’t come frequently to the clinic for drug refills.’ (P07, Female, Healthcare worker, Registered Nurse)

‘So, for adolescents, what we do when they come, we fast-track them. They don’t have to wait in the queue. We quickly identify them and attend to them as soon as possible.’ (P20, Female, Healthcare worker, Registered Nurse, ART In-charge)

Additionally, weekend ART services were made available to further ease access, particularly for in-school youth. Outreach models, including both community- and school-based services, were also employed to reach adolescents in their own environments and to extend the reach of facility-based programmes:

‘We offer services during the weekend. Even on weekends, there are adults who come. But when an adolescent comes on a weekend, they are the priority.’ (P01, Female, Healthcare worker, HIV Nurse Practitioner)

Psychosocial support emerged as a critical component of service delivery in addressing non-biomedical barriers to adherence:

‘And you know, with the adolescents, it involves more of counselling, understanding what their situation, their pressures and what is going to be affecting their adherence compared to the adults, who already know they have to take their medication.’ (P12, Female, Healthcare worker, Medical doctor)

Support groups and educational support programmes were also identified as valuable in promoting engagement and resilience among adolescents. Additionally, targeted support for orphans and vulnerable children was emphasised as essential to mitigating the social and economic challenges that impact treatment adherence and retention in care:

‘There we also have support groups where different adolescents come in with different challenges and they meet at that level, at their peer level and they get to discuss at that level as a peer group.’ (P10, Female, Healthcare worker, Psychosocial Counsellor)

‘An NGO runs a programme that gathers more details about adolescents (vulnerable) and guardians to assess their needs. This programme offers support such as school uniforms, books, or other necessary supplies, which has been an ongoing effort to enhance their well-being.’ (P05, Male, Healthcare worker, Clinical Officer)

#### Theme 1.4: Health workforce capacity building

Capacity building emerged as a key strategy in enhancing the delivery of adolescent-focused HIV services. At the core of these efforts was the development of a national training curriculum, which provided standardised training materials tailored for both HCWs and community-based staff. These training packages were designed to build provider competencies in delivering youth-friendly services:

‘We have the adolescent health training manual that is used to train these staff [*healthcare workers*] who are providing services. We also have the peer adolescent peer support manuals.’ (P30, Male, HIV Programme Manager, Ministry of Health)

In addition to formal training, ongoing mentorship and supportive supervision were highlighted as critical components for reinforcing learning and ensuring quality service provision. On-site mentorship, often conducted by experienced clinical mentors, allowed providers to apply knowledge in real time and receive immediate feedback, thereby strengthening confidence and consistency in adolescent service delivery:

‘Programme officers go round to supervise and provide mentorship to the service providers.’ (P29, Female, HIV Programme Manager, District Health Office)

Taken together, these programmatic responses, characterised by strategic leadership, supportive policy direction and adolescent-focused service delivery models, establish a strong enabling framework for adolescent HIV care; however, participants emphasised that their effectiveness is highly dependent on health system capacity and the broader social context in which services are delivered.

### Thematic domain 2: Implementation challenges

The implementation challenges described by participants reflect structural and individual-level constraints that directly limit the reach, quality and sustainability of the programmatic responses outlined above, highlighting gaps between policy intent and everyday service delivery realities.

Despite efforts to scale-up adolescent-focused HIV services, several implementation challenges were identified at multiple levels of the health system.

#### Theme 2.1: Inadequate infrastructure

Infrastructure constraints were a major impediment to delivering adolescent-friendly care:

‘Another issue is our adolescent-friendly space is very small; it can’t hold more than 10 people.’ (P05, Male, Healthcare worker, Clinical Officer)

Technological limitations such as poor internet connectivity and inadequate hardware undermined digital system performance:

‘We usually have network issues. Sometimes network just drops completely.’ (P06, Female, Healthcare worker, Psychosocial Counsellor)

#### Theme 2.2: Health system constraints

Health workforce challenges were reported as recurrent across sites. Facilities face a shortage of trained HCWs with specific competencies in adolescent care. High staff turnover further erodes service continuity, while few HCWs are dedicated exclusively to adolescent services. In some cases, provider-related stigma and bias negatively influence the care experience for adolescents:

‘It’s mainly the nurses who are trained and the psychosocial counsellors, there are some who are trained. Clinicians and doctors are not usually included in the trainings focused on adolescent services.’ (P16, Female, Healthcare worker, HIV Nurse Practitioner)

Sustainability concerns of adolescent HIV programmes emerged because of donor dependence:

‘These days, we lack fundings. Some time back, we used to have people that were supporting us. For example, we come here on Saturdays, people would go home with something, snacks and the likes, but this time around, no.’ (P06, Male, Healthcare worker, Psychosocial Counsellor)

Supply chain inefficiencies compound service delivery challenges. Frequent stock-outs of essential commodities, including pregnancy test kits, and inadequate transport systems for follow-ups or home visits, constrain the ability to maintain continuity of care:

‘Sometimes we run out of the screening tests such as pregnancy tests, which is a required test for those seeking for family planning services.’ (P09, Female, Healthcare worker, Registered Nurse)

#### Theme 2.3: Individual-level barriers to adherence and engagement

At the individual level, adherence and retention in care were undermined by several behavioural and structural factors. Adolescents were reported to frequently miss weekday appointments or interrupt treatment, often because of competing academic demands, long distances to health facilities or low personal motivation for continued ART:

‘And most adolescents are in school, so Thursday appointments clash with classes – causing missed visits.’ (P05, Male, Healthcare worker, Clinical Officer)

Despite psychosocial support, stigma remained a widespread barrier. Adolescents commonly faced self-stigma, peer pressure and challenges related to non-disclosure of their HIV status to friends and family, all of which compromised their engagement with care:

‘The same stigma, fearing to be stigmatised or maybe by themselves, they have self-stigma.’ (P24, Male, Healthcare worker, Registered Nurse)

‘Some of the challenges we face include peer pressure and poor disclosure.’ (P23, Male, Healthcare worker, Clinical Officer)

Family-related challenges also played a critical role. Issues such as unsupportive caregivers, lack of parental contact or temporary guardianship arrangements disrupted adherence and follow-up:

‘We have some adolescents that are orphans and they are being kept by their guardians and some guardians are not even concerned about their conditions.’ (P17, Female, Healthcare worker, HIV Nurse Practitioner)

‘Others are maybe just staying with guardians who are something else. Others are hiding, maybe the guardian doesn’t know this child is on ARVs, so again, they will not take their medication.’ (P12, Female, Healthcare worker, Medical doctor)

Collectively, these system and individual-level challenges were described as mutually reinforcing, with health system weaknesses often exacerbating stigma, poor adherence and disengagement from care among adolescents.

### Thematic domain 3: Opportunities for improved programmatic response and service delivery

Importantly, the opportunities proposed by HCWs and HIV programme managers emerged directly from these interconnected challenges, illustrating how identified gaps in policy, health systems and service delivery informed context-specific strategies for strengthening adolescent HIV services.

Several key opportunities for improved programmatic response and service delivery for ALHIV were provided.

#### Theme 3.1: Policy

Policy plays a critical role in shaping the delivery of health services for ALHIV, influencing their access to testing, treatment and ongoing care. Human immunodeficiency virus programme managers emphasised the need for policy reform to better support adolescent health outcomes. Specifically, they advocate for lowering the legal age of consent for accessing HIV services, noting that current consent laws often act as barriers to timely diagnosis and treatment initiation for adolescents:

‘I think the biggest one right now that I think affects everything is the age of consent and I think this is something everyone is battling with and we’ve tried advocating for it.’ (P25, Female, HIV Programme Manager, NGO)

#### Theme 3.2: Health system and infrastructure

At the health system and infrastructure level, participants emphasised the need to expand adolescent-friendly spaces through the upgrading of facilities, establishment of adolescent-only clinic areas and creation of additional centres of excellence:

‘So probably it would be to find a bigger space for them. Because sometimes you find they have come in in numbers, you want to have probably a group session, but the room is very small.’ (P21, Female, Healthcare worker, Registered Nurse)

‘I think and suggest separating the main ART and adolescent ART because most of the adolescents, what I’ve come to understand is they are not so comfortable when they are in the midst of elder people.’ (P23, Female, Healthcare worker, HIV Nurse Practitioner)

Strengthening human resource capacity was also highlighted, including training and retaining adolescent-specialised HCWs, assigning dedicated focal persons and sensitising staff to adolescent needs:

‘If all health workers can be put on board through training in adolescent-friendly services to support them, I think that would be a positive move.’ (P17, Female, Healthcare worker, HIV Nurse Practitioner)

In addition, improving logistics and supply chain management, particularly ensuring consistent availability of laboratory commodities and allocating dedicated transport for follow-ups, was considered essential.

#### Theme 3.3: Service delivery

At the service delivery level, participants recommended strengthening adolescent-centred models by scaling up the integration of additional services such as SRH within HIV service delivery and peer-led outreach:

‘The Ministry of Health has come up with a newer initiative that tries to integrate HIV and sexual reproductive health through their new initiative. They’re still yet to implement it.’ (P25, Female, HIV Programme Manager, NGO)

Improving adherence and engagement through strengthened retention packages, adolescent-specific counselling and digital follow-up tools needs to be prioritised:

‘And of course we have different apps coming up to support adolescents.’ (P26, Female, HIV Programme Manager, NGO)

Lastly, supporting family and caregiver engagement by educating guardians and building caregiver support networks was viewed as critical to sustaining adolescent care and treatment outcomes:

‘I honestly feel we’re spending too much money on biomedical interventions. I feel funding should go towards Social Behavioural Communication.’ (P25, Female, HIV Programme Manager, NGO)

These opportunities emphasise the need for coordinated action across policy reform, health system strengthening and adolescent-centred service delivery, underscoring the interdependence of system-level interventions and individual-level support in improving engagement, adherence and retention among ALHIV.

## Discussion of findings

In line with the study aim of exploring how HIV service delivery and support programmes for adolescents are implemented, experienced and perceived by HCWs and HIV programme managers in Lusaka District, our findings highlight three key areas. Firstly, participants described existing programmatic responses characterised by strategic leadership, supportive policy direction, data-driven monitoring, multisectoral collaboration and adolescent-focused service delivery models, including adolescent-friendly services, DSD and psychosocial support. Secondly, they identified substantial implementation challenges at the health system and individual levels, including infrastructural and technological limitations, human resource shortages, funding and supply chain constraints, as well as stigma, poor adherence and caregiver-related barriers. Thirdly, participants proposed actionable opportunities for improvement to strengthen policy frameworks, expand adolescent-friendly infrastructure, build human resource capacity, improve logistics, enhance adolescent-centred service models and promote family and caregiver engagement to improve HIV care and treatment outcomes among ALHIV in this urban setting.

The study found that provider training and ongoing mentorship were critical enablers for the effective delivery of adolescent services.^35^ Standardised training manuals and mentorship programmes helped to build provider competencies and promote consistent service quality. Nonetheless, gaps remained in the coverage and reach of these training efforts, particularly among clinical cadres not directly targeted by current capacity-building initiatives.

Despite notable progress, several cross-cutting challenges undermined the scale and sustainability of adolescent HIV services. The legal age of consent remains a significant bottleneck, limiting adolescents’ independent access to essential SRH and HIV services, a barrier similarly reported in previous studies.^36^ Addressing this policy barrier is crucial to improving timely access, continuity of care and health outcomes for adolescents. Infrastructure limitations, such as insufficient space for adolescent-focused care, hampered the ability to deliver private and engaging services, consistent with earlier research.^37^ Technological constraints, including unreliable internet connectivity and limited digital tools, have further impeded effective data capture and service coordination, echoing findings from prior studies.^38^ Reliance on donor funding impeded continuity of care, as observed in previous research,^39^ while human resource shortages, high staff turnover and limited specialisation in adolescent health constrained the continuity and quality of services in line with earlier reports.^40^ Additionally, supply chain disruptions, particularly stock-outs of essential commodities and limited transport capacity, have created barriers to maintaining consistent care and follow-up as documented in prior studies.^41^

At the individual level, stigma, both internalised and external, remain deterrents to care engagement, in agreement with previous findings.^42,43,44^ Adolescents often face school-related conflicts as noted in earlier research,^45,46,47^ lack adequate caregiver support as reported in a previous study^48^ and experience difficulties in disclosing their HIV status, all of which undermine adherence and retention, in agreement with a previous study.^49^ Collectively, these findings suggest evidence emphasising the multi-dimensional nature of barriers to adolescent HIV care and the need for interventions that are not only biomedical but also social and behavioural in focus. From a research perspective, these findings highlight the need for longitudinal and mixed-methods studies to examine how health system, policy and psychosocial factors interact over time to influence adolescent engagement and outcomes, as well as comparative studies assessing differential effects of adolescent-friendly and community-based models across contexts.

This study offered several actionable opportunities for improved programmatic response and service delivery for ALHIV. These included expanding adolescent-friendly infrastructure, training and retaining adolescent-focused health workers and strengthening logistics systems for service continuity, in line with evidence that differentiated, youth-tailored models improve engagement and retention in care among adolescents in sub-Saharan Africa.^22^ Enhancing peer-led models, integrating SRH with HIV services and decentralising care to the community level were also viewed as critical for expanding access and acceptability. Similar approaches, such as support groups, peer-delivered behavioural interventions, adherence clubs and community ART delivery, have shown positive effects on ART adherence and retention among AYP, although the effect sizes are often modest and context-dependent.^50,51^ Likewise, integration of SRH and HIV services has been associated with improved uptake of HIV testing, contraception and other preventive services, but implementation in many settings remains constrained by vertical programme structures and legal or policy barriers affecting adolescents, which may partly explain variability between our findings and those reported elsewhere.^23,52^ Decentralised and community-based models of adolescent DSD highlight that decentralisation alone does not guarantee better retention without concurrent youth-friendly design and psychosocial support, which may account for differences in effectiveness observed across programmes.^53^ Moreover, supporting families and caregivers through education and structured involvement was identified as key to improving adolescent treatment outcomes and long-term engagement with care. Prioritised investment in behavioural and social interventions was recommended to complement biomedical strategies and address the underlying determinants of poor adherence.^53^ Prioritised investment in behavioural and social interventions was recommended to complement biomedical strategies and address the underlying determinants of poor adherence, echoing systematic review findings that multicomponent interventions combining counselling, mobile phone messaging, peer or lay worker support and community ART delivery achieve better adherence outcomes than standard biomedical care alone.^50^

Future research should also strengthen adolescent participation by foregrounding adolescents’ lived experiences, testing digital and peer-led innovations using robust implementation research designs and generating context-specific evidence on scalability, cost-effectiveness and sustainability of adolescent HIV interventions.

### Strengths and limitations

A key strength of this study lies in its methodological rigour. The use of an inductive content analysis approach enabled findings to emerge directly from participants’ narratives without being constrained by a predetermined theoretical framework. Analytical rigour was strengthened through iterative coding, investigator triangulation and consensus-based development of a comprehensive codebook involving multiple authors. Cross-checking of transcripts against agreed codes and themes further enhanced the credibility and dependability of the analysis. In addition, the inclusion of both HCWs and HIV programme managers enabled triangulation across participant groups, contributing to analytic depth and robustness.

Further, the study provides critical insights into the strengths, gaps and opportunities within the existing system of care for ALHIV, as viewed by HCWs and HIV programme managers. While these findings offer valuable contributions to understanding service delivery, they should be interpreted in light of certain limitations. Firstly, the perspectives presented reflect the views of HCWs and HIV programme managers, which, while valuable, may not fully capture the experiences and needs of ALHIV themselves. Secondly, the study was conducted in specific healthcare settings, which may limit the generalisability of the findings to other regions or contexts with different health system structures, resource availability or sociocultural dynamics. The insights gained are context-specific and may not be directly transferable to all settings without considering local variations. Thirdly, social desirability bias may have influenced some participants’ responses, particularly given their professional roles and involvement in the delivery and management of adolescent HIV services. Participants may have been inclined to present their programmes in a more favourable light or downplay challenges to avoid perceptions of poor performance. Lastly, the IDIs, while rich in detail, represent a snapshot in time. The dynamic nature of HIV programmes and ongoing health system reforms mean that service delivery experiences and perspectives may evolve, potentially impacting the relevance of some findings over time.

### Implications and recommendations

This study highlights the need for multi-level interventions to strengthen adolescent HIV care in Zambia. Policy reforms, such as lowering the age of consent for HIV services, integrating adolescent-specific indicators into the health information system and ensuring sustainable financing, are critical for creating an enabling environment. At the service delivery level, scaling up adolescent-friendly models, integrating HIV care with SRH services and addressing workforce and commodity gaps are essential to improve continuity of care. Strengthening psychosocial support, peer-led interventions and caregiver engagement can further enhance adherence and retention.

Future research should centre adolescents’ perspectives to examine alignment with HCWs and programme managers, evaluate DSD and digital health innovations and generate longitudinal evidence on policy and programme effectiveness, building more responsive adolescent-focused HIV systems.

## Conclusion

This study highlights the importance of adolescent-responsive health systems in addressing the multifaceted barriers that hinder engagement and adherence among ALHIV in Zambia. Strategic leadership, supportive policy direction and tailored service delivery models such as differentiated care, integrated SRH services and youth-friendly spaces play a vital role in improving access and responsiveness. However, persistent structural, technological and human resource challenges, along with stigma and limited family support, continue to compromise the quality and sustainability of care. Strengthening infrastructure, expanding provider training, investing in behavioural and social interventions and enhancing community and caregiver involvement are essential to improving outcomes. To meet the UNAIDS 95-95-95 goals, national HIV responses must continue evolving to deliver holistic, adolescent-centred care.
